# Lipidomic analysis enables prediction of clinical outcomes in burn patients

**DOI:** 10.1038/srep38707

**Published:** 2016-12-16

**Authors:** Peter Qi, Abdikarim Abdullahi, Mile Stanojcic, David Patsouris, Marc G. Jeschke

**Affiliations:** 1 Faculty of Medicine, University of Toronto, Toronto, Canada; 2 Biological Sciences, Sunnybrook Research Institute, Toronto, Canada; 3 Ross Tilley Burn Centre, Sunnybrook Health Sciences Centre, Toronto, Canada; 4 Department of Surgery, Division of Plastic Surgery, Department of Immunology, University of Toronto, Toronto, ON, Canada

## Abstract

Recent discoveries have highlighted the novel metabolic functions of adipose tissue in enhancing hypermetabolism after trauma. As the exact function and expression profiles of serum lipids and free fatty acids (FFA) are essentially unknown, we determined the lipidomic expression profile after burn in correlation to clinical outcomes to identify important lipid mediators affecting post-burn outcomes. We conducted a prospective cohort study with 46 adult burn patients and 5 healthy controls at the Ross Tilley Burn Center in Toronto, Canada. Patients were stratified based on major demographic and clinical variables, including age, burn severity, mortality, and sepsis. Serum FFAs and inflammatory markers were measured during acute hospital stay. We found that FFAs were acutely elevated post-burn and returned to baseline over time. Greater burn severity and age were associated with an impaired acute response in unsaturated FFAs and pro-inflammatory cytokines. Elevations in saturated and mono-unsaturated FFAs correlated significantly to increased mortality. In summary, persistent elevation of unsaturated lipids was associated with a functionally altered inflammatory-immunological milieu and worse clinical outcomes. The present lipidomic analysis indicates profound alterations in the lipid profile after burn by characterizing key lipids as potential diagnostic and outcome indicators in critically injured patients.

Severe burn injury leads to a sustained and detrimental hypermetabolic stress response, characterized by marked substrate mobilization and catabolism^1^,^2^. The exact pathophysiology and mediators of this complex response is not entirely known and cannot be fully explained by post-burn stress alone. Interestingly, recent developments in metabolic research have increasingly focused on adipose tissue metabolism. Others and we have recently shown that the adipose tissue notably plays a more important role in trauma than previously thought^3^,^4^,^5^. In particular, the subcutaneous white adipose tissue undergoes browning post-burn, most likely associated with adrenergic stress^4^,^5^. Accordingly, burn patients exhibit significantly altered fat metabolism, characterized by increased peripheral lipolysis, inadequate hepatic beta-oxidation, and futile cycling of energy substrates, including circulating free fatty acids (FFAs) and triglycerides^6^,^7^. It is important to note, that post-burn lipid hypermetabolism contributes to a state of chronic inflammation characterized by ER stress, hyperglycemia, and impaired organ function due to fatty infiltration^8^,^9^.

Fat metabolism is generally differentiated based on different lipid species. Saturated fatty acids (SFAs) have long been associated with metabolic diseases and inflammation, such as metabolic syndrome and type 2 diabetes (T2DM)^10^. On the contrary, monounsaturated (MuFAs) and polyunsaturated (PuFAs) largely exert beneficial effects in metabolism and inflammation^6^,^11^,^12^. In particular, omega-3 (w-3), omega-6 (w-6) PuFAs, and their derivatives are predominantly derived from exogenous sources and considered essential in promoting and resolving inflammation following trauma by providing a source for resolvins, eicosenoids, and prostaglandins^13^,^14^. Hence, lipids likely also play a crucial role in regulating inflammatory and immune functions after burn. To date only fragments of the post-burn fat metabolic response have been delineated. Most clinical studies only include free fatty acids, plasma triglycerides and cholesterol, which provide a very narrow window of lipid metabolism after burn. Therefore, the aim of the current study was to determine the lipid expression profiles of burn patients through lipidomic analyses, in correlation to metabolism, inflammation and clinical outcome.

Lipidomic analysis is rapidly becoming an important tool in medicine to enable high-throughput characterization of lipid species in biological systems^15^. Clinically, specific serum lipid abnormalities are increasingly used as systemic predictors of T2DM, insulin resistance, and nonalcoholic fatty liver disease^16^,^17^. Similar to these chronic diseases, burn injury is also characterized by insulin resistance, hyperglycemia, and hepatic steatosis, as a consequence of persistent hypermetabolism^1^. Thus, profiling large-scale changes in lipid composition and determining the trajectories of individual lipid species in burn patients may identify currently unknown yet important mediators contributing to poor outcome in burn patients. Here, we hypothesize that burn trauma induces an elevation of all circulating FFAs, associated with higher burn severity, worse clinical outcome, and greater systemic inflammation.

## Results

### Clinical Demographics

A cohort of 46 burn patients with burns over 20% TBSA burn and 5 healthy controls were included in the analysis (Table 1). Patients were on average 49 years of age, predominantly males (59%), with no demographic differences between burn patients and healthy controls. The average burn size was 38% TBSA (total body surface area), with an 24% incidence of inhalation injury. Average length of stay (LOS) was 43 days, which resulted in 1.2 days per percent TBSA. Patients were taken to the OR an average of 4.6 times during their stay. 57% of the patients became septic, 7% had MOF, and 24% of the patients succumb due to the burn injury. The patients were selected based on uncomplicated hospital outcomes or complicated outcomes including sepsis, MOF, and death, allowing lipidomic analysis associated with these outcomes, and are therefore not representative of a randomized prospective enrolment.

### Alterations in FFA in burn patients over time

**Figure 1:**
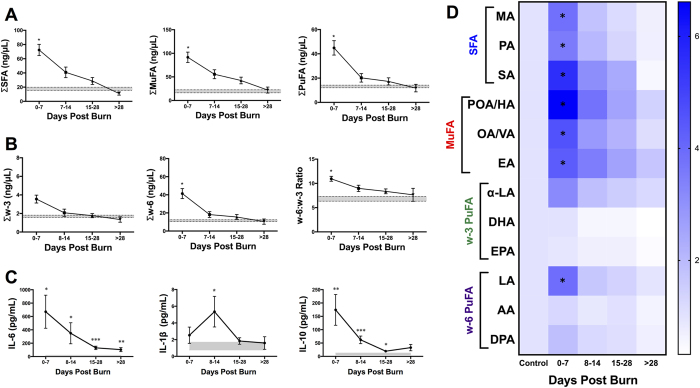
FFA and cytokine alterations in burn patients over time. (**A**) All NEFAs. (**B**) Major FFA species (SFA, MuFA, and PuFA). (**C**) PuFAs sub-species (w3, w6, and w6:3 ratio). (**D**) Pro- and anti-inflammatory cytokines (IL-6, IL-1β, and IL-10). Shaded area represents values for normal controls. All data expressed as mean ± SEM. *Asterisks* indicate significance (p < 0.05) between control and clinical group compared. Double symbols indicate p < 0.01.

Lipid profiling analysis over time revealed a marked increase in the levels of all major FFA species immediately after burn trauma compared to healthy controls (Fig. 1). In the first week post-burn, the sum of all NEFAs was elevated 4.2 fold (p = 0.008; Supplementary Figure 1A). As shown in Fig. 1A, concentrations of SFAs, MuFAs, and PuFAs were increased 4.1 fold (p = 0.014), 4.7 fold (p = 0.020), and 3.4 fold (p = 0.040), respectively. We further subdivided the PuFA species into w-3 and w-6 fatty acids (Fig. 1B). Interestingly, only w-6 PuFAs were significantly elevated post-burn compared to healthy controls (3.6 fold; p < 0.05). The differential elevation of w-6 and w-3 FFAs was reflected in a 1.6 fold increase in the w-6:w-3 ratio (p = 0.037). These dramatic and systemic alternations were not persistent, as all FFA species returned to baseline concentrations gradually over a four-week period. Specifically, when we examined individual FFAs, SFAs, MuFAs, and essential PuFAs all followed this trend (Fig. 1D). We therefore concluded that FFA alterations occurred predominantly during the acute phase after injury.

Given the known inflammatory properties of serum FFAs, we next examined the immune profiles of burn patients over time (Fig. 1C). IL-6 had been linked to increased peripheral lipolysis, and here we observed a significant rise in IL-6 concentration persisting beyond four weeks post-burn, mirroring the serum FFA alterations^18^. IL-1β, a pro-inflammatory cytokine, was acutely elevated in the second week post burn and normalized to baseline levels thereafter. Interestingly, there was an acute rise in the anti-inflammatory cytokine, IL-10, which was sustained up to four weeks after the initial trauma.

### Burn Severity and TBSA

Comparing patients with burns <40% and >40% TBSA, we found similar ages (52 vs 45; NS), gender distribution (56% vs 63% male; NS), and incidence of inhalation injury (52% vs 53%; NS) (Supplementary Table 1). Compared to patients with <40% TBSA burns, patients with >40% TBSA injuries had significantly longer LOS (55 days vs 35 days; p = 0.006) and underwent more surgeries (5.8 vs 3.7; p = 0.005).

**Figure 2:**
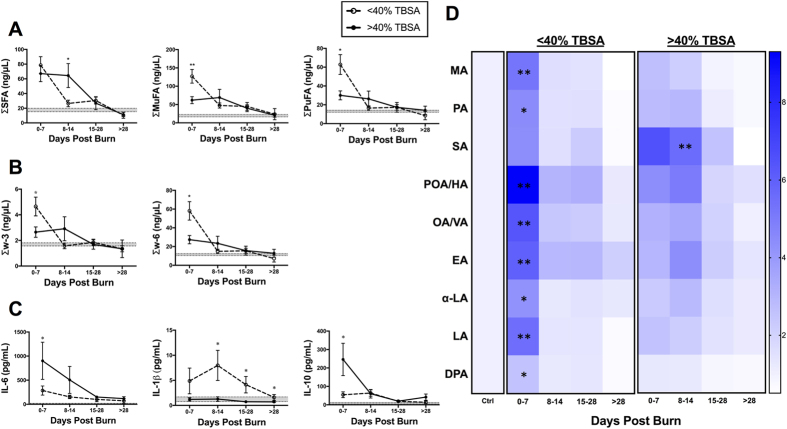
FFA time course stratified based on TBSA (<40% vs. >40%). (**A**) Major FFA species (SFA, MuFA, and PuFA). (**B**) PuFAs sub-species (w3, w6, and w6:w3 ratio). (**C**) Pro- and anti-inflammatory cytokines (IL-6, IL-1β, and IL-10). Shaded area represents values for normal controls. All data expressed as mean ± SEM. *Asterisks* indicates significance (p < 0.05) between <40% vs. > 40% TBSA.

Stratifications of patients based on burn severity revealed unique alterations in the major FFA sub-groups (Fig. 2A). In the first week, the <40% TBSA group had significantly higher unsaturated FFA concentrations, compared to the severe burn injury group, as shown in Fig. 2A and B. This overall difference was explained by a greater increase in MuFAs (palmitoleic, cis-7-hexadecenoic, oleic, vaccenic, and eicosenoic acid) and PuFAs (linoleic acid and α-linolenic acid) in the <40% TBSA group (Fig. 2A and D). Although myristic acid was significantly higher in the <40% group acutely post-burn (p = 0.006), there was no overall differentiation of SFA levels in the first week based on TBSA (Fig. 2D). By the second week, FFA levels were significantly higher in the >40% TBSA group (Supplementary Figure 1A). Unlike the acute post-injury phase, this signal was explained by a persistent elevation of SFAs (p = 0.010), particularly stearic acid (p = 0.007), in more severely burned patients (Fig. 2A and D). Notably, severely burned patients demonstrate an inverse lipid inflammatory response, characterized by a sustained pro-inflammatory lipid response, in the absence of early anti-inflammatory activation of PuFAs.

We then assessed the cytokine profile of the study sample to further delineate this inverse inflammatory response. IL-6 was significantly increased in burns over 40% TBSA burn when compared to <40% TBSA burn. Surprisingly, IL-1β, a well-characterized pro-inflammatory marker, showed a sustained and significant elevation in the <40% TBSA group, with no changes observed in the >40% TBSA group (Fig. 2C). In contrast, the well-established anti-inflammatory cytokine, IL-10, showed an acute and transient elevation in severely burned patients, which was absent in the <40% TBSA group (Fig. 2C).

### Survival

Next, we compared the lipid profiles of non-survivors and survivors of burn injury (Supplementary Table 1). Those who succumbed to their injuries were on average older than the survivors (64 vs 45 years, p = 0.003), but had similar gender distributions (66% vs 36% male; p = 0.085), and no differences in TBSA (38% vs 36%; p = 0.703), LOS (47 days vs 32 days; p = 0.075), incidence of inhalation injury (46% vs 73%; p = 0.118), sepsis (51% vs 73%; p = 0.214), or MOF (3% vs 18%; p = 0.073).

**Figure 3:**
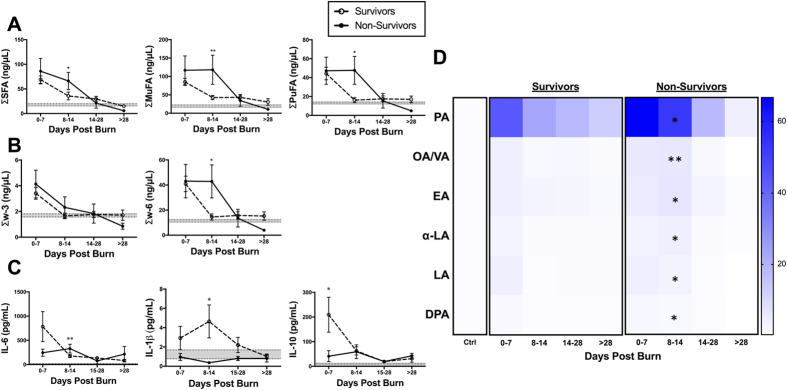
FFA time course stratified based on mortality (survivors vs. non-survivors). (**A**) Major FFA species (SFA, MuFA, and PuFA). (**B**) PuFAs sub-species (w3, w6, and w6:w3 ratio). (**C**) Pro- and anti-inflammatory cytokines (IL-6, IL-1β, and IL-10). Shaded area represents values for normal controls. All data expressed as mean ± SEM. *Asterisks* indicate significance (p < 0.05) between survivors and non-survivors.

Overall, FFA concentrations were not different between survivors and non-survivors acutely post-burn. However, we observed that FFA levels were persistently and significantly elevated in non-survivors compared to healthy controls, attributable to SFAs, MuFAs, and w-6 PuFAs (p = 0.035, 0.028, and 0.007, respectively; Fig. 3A). Particularly, we detected signals for palmitic, oleic/vaccenic, eicosenoic, α-linoelic, linolenic, and doscosapentaneoic acids (Fig. 3D). In short, the pro-inflammatory lipid profile (SFA and w-6 PuFAs) remained elevated in non-survivors after the acute phase, which was absent in survivors. (Fig. 3A and D).

As higher SFA and w-6 PuFA levels have been associated with a greater pro-inflammatory state, we assessed the immune profile of these two groups. In contrast to the lipidomic data, survivors demonstrated a greater and more sustained pro-inflammatory response, as shown by IL-6 and IL-1β, with signals of early anti-inflammatory activation (IL-10), compared to non-survivors (Fig. 3C). These findings suggest that poor clinical outcomes after burn is likely mediated by a failure to mount an appropriate immune response, despite sustained release of pro-inflammatory serum lipids (SFAs and w-6 PuFAs).

### Age

**Figure 4:**
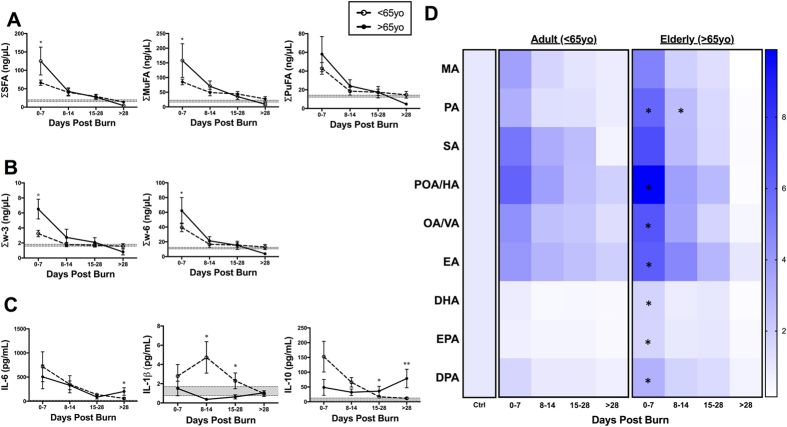
FFA time course stratified based on age (<65yo vs. >65yo). (**A**) Major FFA species (SFA, MuFA, and PuFA). (**B**) PuFAs sub-species (w3, w6, and w6:w3 ratio). (**C**) Pro- and anti-inflammatory cytokines (IL-6, IL-1β, and IL-10). Shaded area represents values for normal controls. All data expressed as mean ± SEM. *Asterisks* indicate significance (p < 0.05) between adult vs elderly. Double symbols indicate p < 0.01.

Elderly patients (>65 yo) had higher rates of mortality (53.8% vs 13.8%; p < 0.01), but significantly lower TBSA (30.3% ± 2.8% vs 40.7% ± 2.5%; p = 0.02), compared to adults (<65 yo; Supplementary Figure 1). Overall, elderly patients showed significantly elevated levels of all FFAs (p = 0.008), particularly SFAs (p = 0.006) and MuFAs (p = 0.016), when compared to healthy controls in the first week post-burn (0–7 days; Fig. 4A). Further analysis revealed that elderly patients had a persistently elevated pro-inflammatory lipid profile, as evidenced by higher palmitic acid levels compared to adult burn patients (Fig. 4D). Interestingly, elderly patients also had elevated levels of anti-inflammatory w-3 lipids during the acute phase (Fig. 4B). Specifically, DHA, a conditionally essential w-3 PuFA, was acutely elevated post-burn in the elderly population, compared to adults (p = 0.008; Fig. 4D). In support of this anti-inflammatory activation, elderly patients had a depressed IL-1β immune profile with a concomitant delayed elevation of IL-10 late into the injury, suggesting a delayed immune response likely facilitated by anti-inflammatory lipid mediators released early post-burn (Fig. 4C). Thus, the present findings are consistent and extend to previous studies supporting a delayed metabolic and inflammatory response to burn injury in the elderly^19^,^20^.

### Sepsis

Next, we stratified the sample into septic and non-septic patients (Supplementary Table 1). Although we found no demographic differences, there was a higher prevalence of inhalation injuries in septic patients (92% vs 8%; p < 0.0001), and they also underwent more surgeries (5.5 ± 0.5 vs 3.5 ± 0.5; p = 0.008) compared to non-septic patients. We found no differences in the TBSA between septic and non-septic patients.

There were no significant differences between septic and non-septic patients for all the major FFA species at any of the time points (Supplementary Figure 2A). There was, however, a significant difference in the C16:1 and C20:1 MuFAs in the first week post-burn (Supplementary Figure 2C). Palmitoleic acid, a C16:1 MuFA hepatically metabolized from palmitic acid, and its isomer, cis-7-hexadecnoic acid were higher in non-septic patients compared to septic patients (p = 0.09) and healthy controls (p = 0.006). Similarly, non-septic patients had significantly higher levels of eicosenoic acid compared to healthy controls (p = 0.020), but not septic patients. In contrast, though non-survivors had persistent elevation of eicosenoic acid, we found no mortality-based differences in C16:1 MuFA concentrations.

### Correlation to Outcome Variables

Adjusting for age, TBSA, and days post-burn, we found that higher risk of mortality was significantly correlated to increases in SFAs (odds ratio 1.06 [95% CI 1.00–1.03]; p = 0.0408) and MuFAs (odds ratio 1.06 [95% CI 1.01–1.11]; p = 0.0285; Table 2). In particular, palmitic and oleic/vaccenic acid, individual FFAs found at higher concentrations in non-survivors, were associated with a 18% (odds ratio 1.18 [95% CI = 1.03–1.35]; p = 0.0186) and 9% (odds ratio 1.09 [95% CI = 1.01–1.17];p = 0.0334) increased risk for mortality, respectively, with every 10 μg/mL increase in serum concentration. Though PuFAs were also elevated in non-survivors, there was no significant association between mortality and serum concentrations of PuFA, w-3, nor w-6 FFAs. Finally, septic status was not significantly correlated to any lipids in this risk-adjusted analysis.

## Discussion

Our results demonstrate that severe thermal injury induces peripheral lipolysis and FFA mobilization, resulting in an acute, global, and complex increase in serum FFAs, which corroborates a number of past observations in burn and trauma populations^3^,^6^,^7^. Recent research has shown morphological remodelling of adipose tissue through browning after burn injury^4^,^5^. Likewise, brown adipose tissue is known to exert an autocrine effect by augmenting systemic lipolysis and FFA oxidation^21^. The role of fat browning in regulating lipid metabolism further cements the importance of the adipose tissue in post-burn hypermetabolism. Paradoxically, serum FFA concentrations following burn injury have been found to be highly variable. Previous studies of serum FFAs in burn patients have focused on total fatty acid content, including fatty acids bound in triglycerides and phospholipids, with reports of decreased lipids early post-burn^22^,^23^. Other studies have reported elevated circulating FFA levels after burn injury or trauma, but lacked uniform and consistent signals over time^8^. In short, no standard and reliable patterns of post-burn serum FFAs have been reported before the current study. Consequently, the connection between post-burn fatty acid metabolism and patient outcome has remained elusive till now despite growing consensus of the significance of post-burn lipid metabolism.

Here we present the discovery and validation of serum lipid changes that distinguish not only healthy individuals from burn patients, but also specific populations of burn patients that will progress to have poor clinical outcome during hospitalization. We have also identified lipid profile features that have metabolic and immunological correlations in the response to burn injury. There is an increasing recognition that various FFA species exert differential physiological effects^24^. A key finding of our study was that elevations in SFAs were associated with higher burn severity, mortality, and a suppressed pro-inflammatory profile beyond the acute phase of trauma. In contrast, early PuFA elevations were associated with a delayed immune response in elderly burned patients, consistent with our previous findings^19^,^20^.

Studies have previously associated SFAs with metabolic diseases (Table 3), including T2DM and obesity, by inducing insulin resistance, ER stress, and inflammation^10^,^20^,^25^. In fact, serum palmitic acid concentration remained elevated beyond the acute phase, and correlated to increased mortality irrespective of age, burn severity, or outcome. Moreover, greater burn severity was associated with higher IL-6, consistent with previous studies^26^. We and other recently demonstrated that IL-6 is necessary for browning of white adipose tissue in burn, likely through adrenergic activation of peripheral lipolysis^5^. Surprisingly, greater burn severity was also associated with an attenuated pro-inflammatory response, despite persistent SFA elevation. We hypothesize that IL-6 induces preferential mobilization of saturated FFAs over unsaturated lipid species, which are important components in the inflammatory cascade. Alternatively, we recently found that chronic exposure to palmitate induces M2 macrophage differentiation in adipose tissue, which dampens inflammation and facilitates tissue repair^27^. Therefore, SFAs may in fact contribute to anti-inflammatory activation in select clinical contexts, contrary to its long-established role as a pro-inflammatory lipid species. Indeed, we observed anti-inflammatory activation (IL-10) in the >40% TSBA group in the absence of anti-inflammatory PuFA recruitment. Together, our data imply that palmitic acid may serve to dampen inflammation through macrophage polarization leading to increased mortality, suggesting a key role for early pro-inflammatory responses in improving survival.

Unlike SFAs, dietary MuFAs have been shown to decrease insulin resistance, facilitate wound healing, and protect cardiovascular health^12^,^28^. MuFAs originate from both dietary sources and hepatic desaturation reactions, and provide an excellent source for energy (Table 3). Interestingly, MuFAs were acutely elevated in selective clinical sub-groups, namely those with lower burn severity, age, and no sepsis. Therefore, severe burns may potentially impair the acute mobilization and synthesis of MuFAs, which predispose patients to adverse clinical outcomes. Contrary to this theory, non-survivors had a persistent elevation of MuFAs accounted for by differential elevation of oleic and vaccenic acids. Notably, we also correlated oleic and vaccenic acids to an increased risk of mortality, in contrast to their known beneficial physiologic roles^12^. Thus, we believe that MuFAs play an important role in the acute phase by satisfying the increased metabolic demands post-burn. However, their continued presence may impede recovery from traumatic injuries.

In the context of burn injury, the role for PuFAs is undoubtedly the regulation of inflammation and the immune response (Table 3). Generally, w-6 PuFAs promote and mediate pain and inflammation, while w-3 PuFA derivatives aid in the resolution of inflammation and dampen the immune response^11^,^29^. In accordance with this model of inflammation, acute elevations of w-6 PuFAs were greater than w-3 PuFAs in the overall burn population. Furthermore, we showed that attenuation of PuFA release correlated to an immunosuppressed cytokine profile in more severe burn injuries. Alternatively, a subset of PuFA (w-3 and w-6) derivatives, termed specialized pro-resolving mediators (SPMs), are synthesized early in the inflammatory response and pave the road for the eventual resolution of inflammation^30^. SPMs have been shown to enhance neutrophil migration and decrease post-burn mortality in animal models^31^. We observed a persistent SFA and w-6 PuFA elevation in non-survivors without a corresponding pro-inflammatory serum profile, which may be attributable to the alternative activation of the w-6 SPM pathway. Indeed, a similar profile was observed in elderly patients. Elderly burn patients had elevated levels of conditionally essential w-6 and w-3 PuFAs during the acute phase, suggestive of an up-regulation of PuFA metabolism converting essential PuFAs to their conditionally essential counterparts. Furthermore, we found persistently anti-inflammatory immune environment in the elderly, likely a consequence of excessive early SPM activation. Whether the SPM expression profile will correlate to their PuFA precursors remains to be determined. Indeed, we found no correlation between septic status and PuFA levels in our cohort of patients. Notably, C16:1 MuFAs was acutely suppressed in septic patients early post-burn, which may be explained by hepatic dysfunction in burn injury^32^. Hence, we believe that though PuFAs modulate and contribute to an altered immune environment, they are neither sufficient nor necessary to cause infection and/or sepsis, which are complex processes mediated by multiple organ dysfunction^33^. Regardless, these findings suggest that in addition to dampening inflammation, the premature activation of SPMs may impair the host’s defense to mount an adequate immune response.

Current therapeutic strategies to counteract acute hypermetabolic stress focus on beta-blockade with propranolol, which acts partly by reducing peripheral lipolysis^34^. Interestingly, propranolol also improves insulin sensitivity and decreases hyperglycemia in burn patients, likely by reducing pro-inflammatory fatty acids in circulation^3^,^35^. Although there are few adverse events associated with propranolol, propranolol administration in murine models has been shown to attenuate immune responses and increases mortality during sepsis^35^. The current study clearly demonstrates that different clinical trajectories are associated with unique serum FFA profiles. Non-specific beta-blockade may unintentionally disrupt appropriate physiologic lipid responses to trauma. Alternatively, dietary supplementary of w-3 PuFAs have been shown to decrease pro-inflammatory cytokines (e.g. IL-1 and TNF-α) in animal and humans, by reducing arachidonic acid-derived inflammatory eicosanoids^36^,^37^. In contrast, typical Western diets contain greater proportions of w-6 over w-3 PuFAs, which has been postulated to promote inflammation and metabolic syndrome^38^. Moreover, the true impact of dietary lipid supplementation is likely more accurately represented by membrane phospholipid compositions, which requires long-term interventions^23^,^39^. To achieve better clinical outcomes, therefore, a combination of beta-blockade and specific nutritional interventions at appropriate time points may ultimately improve patient outcome and prevent complications in burn injury.

To our knowledge, this is the first published report of a blood-based lipidomic analysis performed in burn patients. Our lipidomic metabolic panel identifies specific cohorts of burn patients who are at risk for chronic hypermetabolism as well as poor outcome, based on clinical and demographic variables. Our findings also challenge the current approach of separating lipid species as “good” or “bad” fat, by presenting a more nuanced perspective of the role of lipids in inflammation and trauma. Ultimately, more comprehensive lipidomic analyses will have to be tested in randomized controlled trials with larger sample sizes to evaluate the potential utility of candidate lipid biomarkers in burn patients with regards to predicting outcomes. As blood samples are easier and less costly to obtain, this should facilitate screening in large-scale clinical trials for future clinical use in the burn population. We thus consider our findings a major step toward in the development of novel diagnostic and therapeutic strategies for burn patients.

## Methods

### Subjects

A cohort of 46 adult patients with second- and third-degree burns greater than 10% TBSA admitted to our burn center was included in this analysis. All patients in the current study were resuscitated and treated at the Ross Tilley Burn Center according to standard protocols^40^. All patients received continuous enteral nutritional support with early initiation (within 12 hours) via nasogastric, naso-duodenal/naso-jejunal, or by mouth. Enteral feeding consisted of a high-protein, high-glucose, low-fat diet (Isosource HN) and was only interrupted for ORs, with patients NPO (nil per os) for a maximum of six hours. The feeding rate is based on resting energy expenditure (REE) measurements and calculated by the Toronto prediction. We add a stress factor of 1.4 to our calculation. Informed consent was obtained from every patient for blood collection. The study was approved by the Research Ethics Board of Sunnybrook Health Sciences Centre (Study #194–2010), and carried out in accordance with the institutional guidelines.

Patient demographic (age, date of burn and admission, gender, and burn size) and clinical data, complications, and mortality were all prospectively collected. The clinical complications presently reported (sepsis and multiple organ failure/MOF) were determined based on previously established clinical criteria according to the American Burn Association^41^. Patients were divided into subsets based on age (<65 yo vs >65 yo), gender (male vs female), total body surface area (TBSA) of burn (<40% vs >40%), outcome (survival vs death), septic status (septic vs non-septic), and MOF status (MOF vs no MOF). Age stratification was based on definitions from the National Institute of Health and the World Health Organization (WHO). Patients were stratified at 40% TBSA as major catabolic changes have mainly been found at >40% TBSA^42^. Subsequent fatty acid analyses were based on the clinical population as a whole, as well as the stratified sub-groups.

### Serum Sample Collection

Blood samples were collected at admission and subsequently throughout the hospital stay, during early excision and grafting, and subsequent procedures; samples collected from day 0 and day 54 were used for the current study. Venous blood was drawn in collection tubes containing clot activator and gel and centrifuged for 10 min at 1320 rpm; the serum was taken from the supernatant and stored at −80 °C until assayed. Samples were stratified based on the date of collection into 4 groups: 0–7 days, 8–14 days, 15–28 days, and >28 days post-burn.

### GC/MS

Serum fatty acids from patients were analyzed by gas chromatography–mass spectrometry (GC–MS) performed by the Analytical Facility for Bioactive Molecules (AFBM) platform at the Hospital for Sick Children, Toronto, Ontario, Canada. Briefly, serum samples (20 μL) were spiked with an internal standard mix and acidified with HCl. Non-esterified fatty acids were acidified and double-extracted with hexane. The fatty acids were then converted to their pentafluorobenzyl esters using 1% pentafluorobenzyl bromide/diisopropylamine (1:1) and separated by automated gas chromatography (GC Agilent 7890 A, Agilent Technologies, Santa Clara, CA, USA) on a fused-silica SP2380 capillary column (30 m × 0.25 mm × 0.2 μm film thickness; Supelco Analytical, Bellefonte, PA, USA). Fatty acid ions were detected and measured using a MSD Agilent 5975 C quadrupole mass detector (Agilent Technologies, Santa Clara, CA, US). Peaks of fatty acid esters were identified by comparisons with individual fatty acid standards (Supelco Analytical, Bellefonte, PA, USA). Individual fatty acid concentrations (ng/μL) were calculated from the area peak of the internal standards using the Agilent ChemStation software (Agilent Technologies, Santa Clara, CA, US). Saturated (SFA), monounsaturated (MuFA), or polyunsaturated (PuFA) fatty acid contents were calculated by combining the appropriate individual fatty acids into their respective classes (SFA- myrisitic/C14:0/MA, palmitic/C16:0/PA, and stearic acid/C18:0/SA; MuFA- palmitoleic/C16:1 n-7/POA, cis-7-hexadecenoic/C16:1 n-9/HA, oleic/C18:1 n-9/OA, vaccenic/C18:1 n-7/VA, and eicosenoic/C20:1 n-7/EA; PuFA- linoleic/C18:2/LA, α-linolenic/C18:3/α-LA, arachidonic/C20:4/AA), eicosapentaenoic/C20:5/EPA, docosapentaenoic/C22:5/DPA, and docosahexaenoic/C22:6 acid/DHA).

### Multiplex Cytokine Analysis

Three inflammatory cytokines (IL-6, IL-1β, IL-10) were analyzed using the Multiplex platform (Millipore, MA) according to the manufacturer’s protocol. Raw data were processed using Millipore Analyst software. Cytokine concentrations are denoted as mean ± SEM, expressed in pg/mL.

### Statistics

Student’s t-test and one-way ANOVA with Tukey post hoc analysis were used where appropriate. Data were represented as absolute values or mean ± SEM. We compared categorical outcomes using the χ^2^ test. We calculated odds ratios (ORs) for the association of increased serum FFA concentrations with mortality and sepsis, adjusting for age, gender, TBSA, and day post-burn. Significance was established at p < 0.05.

## Additional Information

**How to cite this article**: Qi, P. *et al*. Lipidomic analysis enables prediction of clinical outcomes in burn patients. *Sci. Rep.* **6**, 38707; doi: 10.1038/srep38707 (2016).

**Publisher's note:** Springer Nature remains neutral with regard to jurisdictional claims in published maps and institutional affiliations.

## Supplementary Material

Supplementary Information

## Figures and Tables

**Table 1:** Overall Patient Demographics.

Sample Size, n	Patients	Healthy Control	p-value
	46	5	n/a
Age, mean (years)	49 ± 3	30 ± 3	NS
Sex, male, n (%)	27 (59%)	4 (80%)	NS
TBSA (%)	38 ± 2	n/a	n/a
ORs (n)	4.6 ± 0.4		
LOS (days)	43 ± 4		
LOS/TBSA (Days/%)	1.2 ± 0.1		
Inhalation injury, n (%)	24 (52%)		
Sepsis, n (%)	26 (57%)		
MOF, n (%)	3 (7%)		
Mortality, n (%)	11 (24%)		

TBSA = total body surface area; OR = operations; LOS = length of stay; MOF = multi-organ failure. Data presented as mean ± SEM or percentages.

**Table 2:** Adjusted Odds Ratios for Outcome Variables.

FFA Species	Odds Ratio^a^	95% CI^b^	p-value^c^
Mortality			
NEFA	1.02	1.01–1.05	0.043
SFA	1.06	1.01–1.13	0.041
Palmitic Acid	1.18	1.03–1.35	0.019
MuFA	1.06	1.01–1.11	0.029
Oleic & Vaccenic Acid	1.09	1.01–1.17	0.033
PuFA	1.08	0.96–1.21	0.180
w-3	1.10	0.94–1.29	0.234
w-6	1.09	0.96–1.23	0.181
Sepsis			
NEFA	1.00	0.99–1.01	0.293
SFA	1.01	0.99–1.02	0.277
MuFA	1.01	0.99–1.03	0.327
PuFA	1.02	0.99–1.05	0.293
w-3	1.04	0.97–1.12	0.307
w-6	1.02	0.99–1.05	0.291

^a^Odds Ratio (OR) for all FFA species is computed for every 10 μg/mL increase in FFA concentration, and adjusted for age, gender, TBSA, and day post-burn.

^b^95% Confidence Interval.

^c^Significance is established at p < 0.05.

**Table 3:** Summary of immune functions of free fatty acids.

	Previous studies	Lipidomic analysis in burn patients
SFA	➢Contribute to metabolic diseases (T2DM and obesity)^10^.➢Induces insulin resistance, ER stress, and inflammation^20^,^25^.	➢Correlate to greater age, burn severity, and mortality.➢May have anti-inflammatory roles (e.g. palmitate).
MuFA	➢Cardioprotective, reduces insulin resistance, and improves wound healing^12^,^28^	➢Early mobilization may be physiological and beneficial.➢Persistent elevation correlated to increased mortality.
w-3 PuFA	➢Anti-inflammatory and immune resolution^11^.➢Precursors to special pro-resolving mediators (SPM), i.e. resolvins^30^.	➢Variable effect on inflammation.➢Pro-inflammatory responses from w-6 derivatives.
w-6 PuFA	➢Mediates inflammation and pain^29^.➢Precursors to special pro-resolving mediators (SPM), i.e. lipoxins^30^.	➢Anti-inflammatory effect potentially mediated via SPMs.➢Not necessary/sufficient for sepsis.
