# Supplementary material for: Lipidomic analysis enables prediction of clinical outcomes in burn patients
Source: Sci Rep. 2016 Dec 16;6:38707. doi: 10.1038/srep38707 (PMC5159901; doi:10.1038/srep38707)
Supplement: Supplementary Information [file srep38707-s1.pdf]

Supplementary Information

SREP-16-28340A: Qi, P, Abdullahi, A, Stanojcic, M, Patsouris, D, and Jeschke, MG. “Lipidomic analysis enables prediction of clinical outcomes in burn patients”.

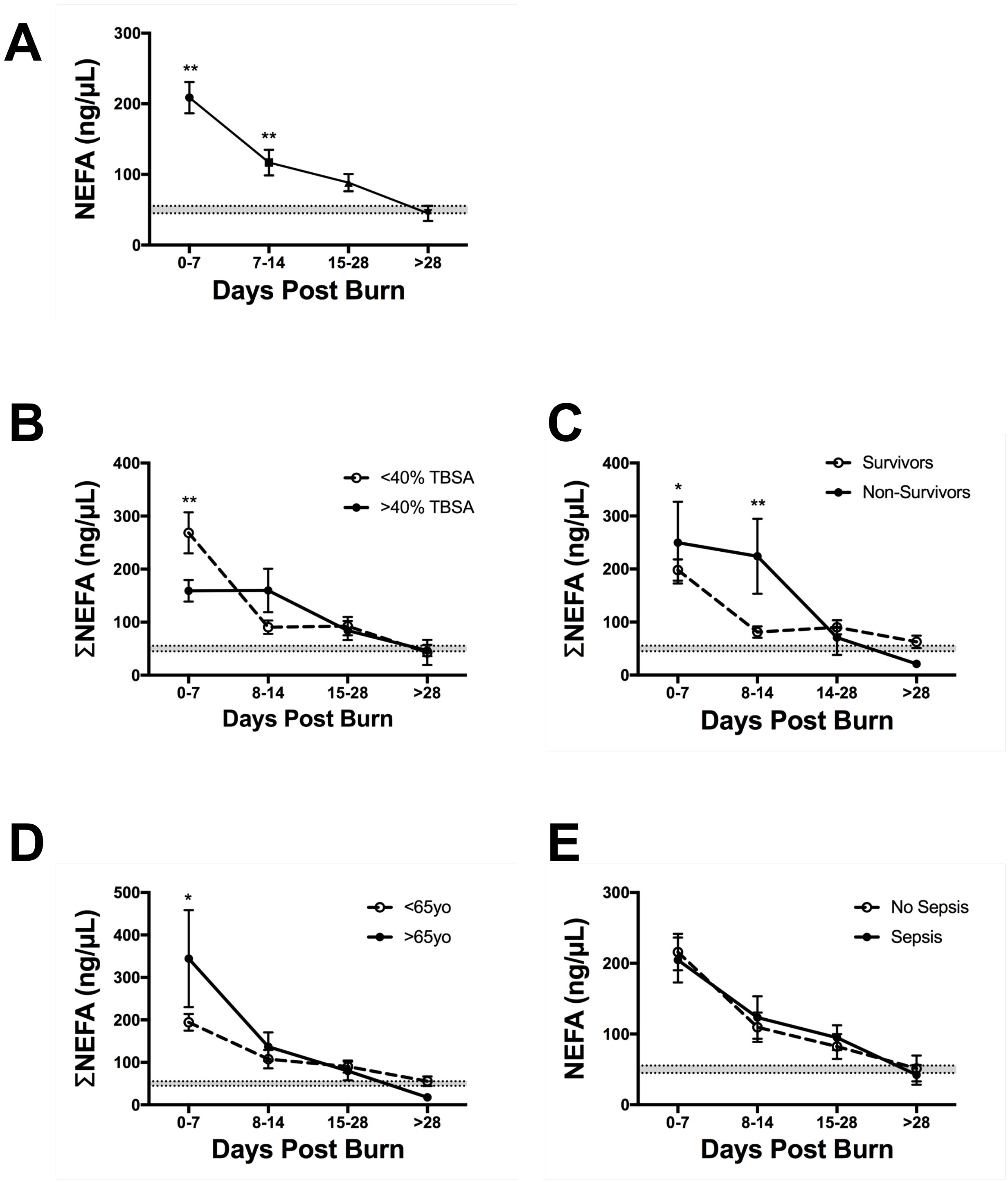

Supplementary Figure 1: Total NEFA concentration in burn patients over time stratified based on **A.** TBSA (<40% vs. >40%), **B.** mortality (survivors vs. non-survivors), **C.** Age (<65yo vs. >65yo), and **D.** Sepsis (Septic vs non-septic). Shaded area represents values for normal controls. All data expressed as mean ± SEM. *Asterisks* indicate significance (p<0.05) between the stratified groups.

Qi, P, Abdullahi, A, Stanojcic, M, Patsouris, D, and Jeschke, MG.“Lipidomic analysis enables prediction of clinical outcomes in burn patients”.

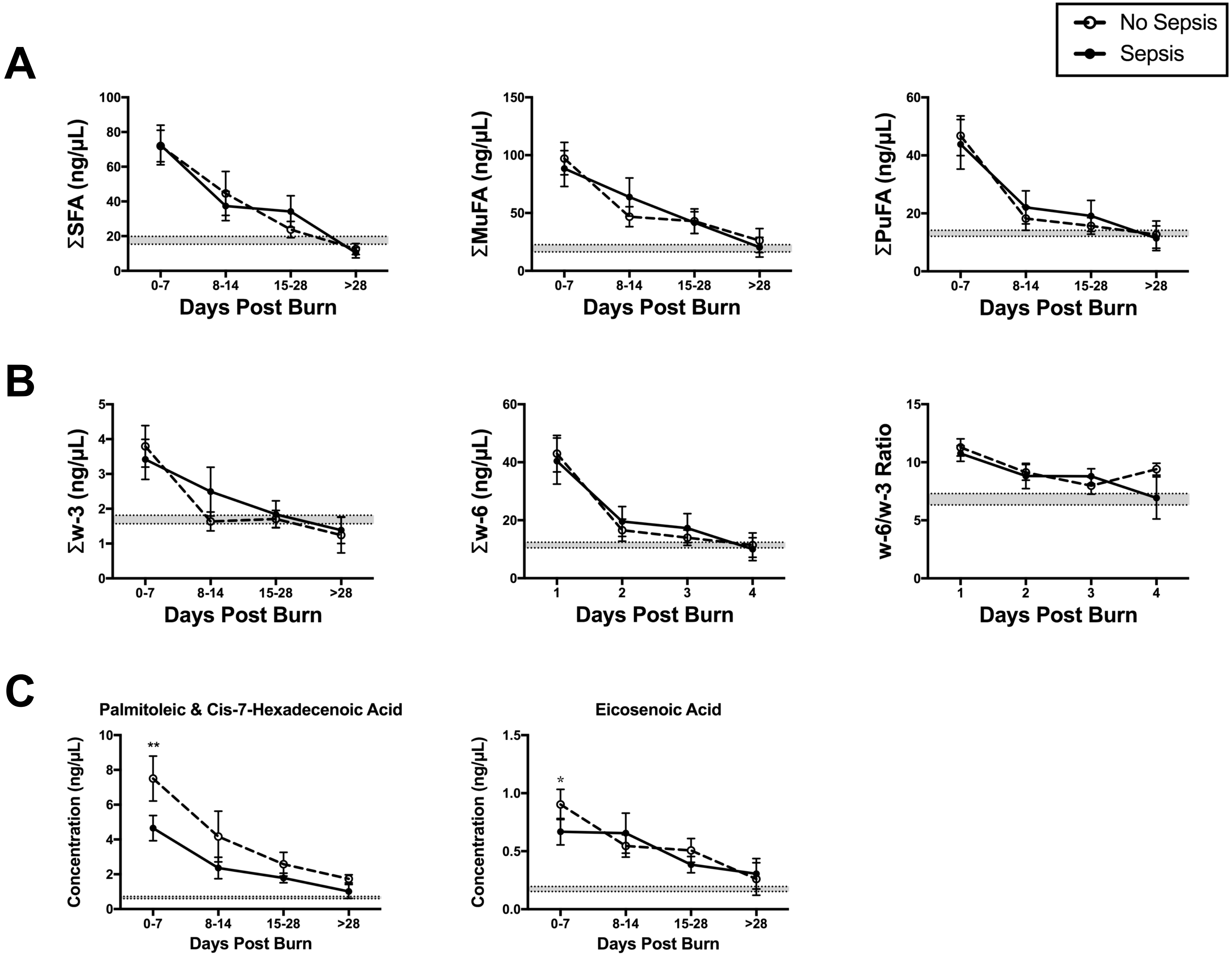

Supplementary Figure 2: FFA time course stratified based on septic status (septic vs non-septic). **A.** Major FFA species (SFA, MuFA, and PuFA). **B.** PuFAs sub-species (w-3, w-6, and w6:w3 ratio). **C.** Individual MuFAs (palmitoleic & cis-7-hexadecenoic, and eicosenoic acids). All data expressed as mean ± SEM. *Asterisks* indicate significance ($p<0.05$) between adult vs elderly. Double symbols indicate $p < 0.01$.

Qi, P, Abdullahi, A, Stanojcic, M, Patsouris, D, and Jeschke, MG.“Lipidomic analysis enables prediction of clinical outcomes in burn patients”.

Supplementary Table 1: Stratified Patient Demographics

	Age			TBSA			Survival			Sepsis		
	<65yo	>65yo	p-value	<40%	>40%	p-value	Lived	Died	p-value	Yes	No	p-value
Sample size, n	33 (72%)	13 (28%)	n/a	27 (59 %)	19 (41 %)	n/a	35 (76 %)	11 (24 %)	n/a	26 (57 %)	20 (44 %)	n/a
Sex, male, n (%)	22 (67%)	5 (39%)	0.08	15 (56%)	12 (63%)	0.606	23 (66%)	4 (36%)	0.085	16 (62%)	11 (55 %)	0.655
Age, mean (years)	40 ± 3	72 ± 2	<0.001	52 ± 4	45 ± 4	0.025	45 ± 3	64 ± 5	0.003	50. ± 4	48 ± 5	0.373
Total TBSA (%)	41 ± 3	30 ± 3	0.02	28 ±1	51 ± 2	<0.0001	38 ± 2	37 ± 4	0.749	38 ± 3	37 ± 3	0.736
LOS (days)	45 ± 5	39 ± 5	0.432	35 ± 3	55 ± 7	0.006	47 ± 4	32 ± 6	0.075	48 ± 5	36 ± 6	0.105
LOS/TBSA (Days/%)	1.1 ± 0.1	1.4 ± 0.2	0.222	1.3 ± 0.1	1.1 ± 0.1	0.279	1.2 ± 0.1	1.0 ± 0.3	0.361	1.3 ± 0.1	1.0 ± 0.1	0.105
Number of ORs, average (n)	4.8 ± 0.5	4.0 ± 0.5	0.341	3.7 ± 0.4	5.8 ± 0.7	0.005	4.9 ± 0.5	3.6 ± 0.5	0.128	5.5 ± 0.5	3.5 ± 0.5	0.008
Inhalation injury, n (%)	18 (55%)	6 (46%)	0.608	14 (52%)	10 (53%)	0.958	16 (46%)	8 (73%)	0.118	24 (92%)	2 (8%)	<0.0001
Sepsis, n (%)	20 (39%)	6 (46 %)	0.373	15 (56%)	11 (58%)	0.875	18 (51%)	8 (73%)	0.214	26 (100%)	0 (0%)	n/a
MOF, n (%)	2 (6%)	1 (8 %)	0.840	1 (4%)	2 (11%)	0.356	1 (3%)	2 (18%)	0.073	3 (12%)	0 (0%)	0.116
Mortality, n (%)	4 (12%)	7 (54 %)	0.003	7 (26%)	4 (21 %)	0.703	0 (0 %)	11 (100 %)	n/a	8 (31%)	3 (15 %)	0.214

TBSA = total body surface area; OR = operations; LOS = length of stay; MOF = multi-organ failure. Data presented as mean ± SEM or percentages.
